# Influence of Systemic Administration of Coq10 Nanoparticles on Ischemia-Reperfusion Injury on Ovaries in Rat

**DOI:** 10.1155/2021/2303417

**Published:** 2021-07-23

**Authors:** Pouria Honardoust, Alireza Najafpour, Rahim Mohammadi

**Affiliations:** ^1^Department of Clinical Sciences, Urmia Branch, Islamic Azad University, Urmia, Iran; ^2^Department of Surgery and Diagnostic Imaging, Urmia University, Urmia, Iran

## Abstract

Using a rat ovary model, effects of COQ10 nanoparticles (NCOQ10) were studied on ischemia-reperfusion injury. In the present experimental study, following randomization of thirty healthy female Wistar rats ∼250 g, the animals were subjected to five experimental groups (*n* = 6): group SHAM : only laparotomy was performed, group IS: only a 3-hour ischemia was performed, group IS/REP: the procedure included a 3-hour ischemia followed by a 3-hour reperfusion, and 50 *µ*L soybean oil (solvent of NCOQ10) was administered 30 min before cessation of reperfusion, group IS/NCOQ10: the procedure included a 3-hour ischemia only and 50 *µ*L (0.3 mmol/lit/IP) of NCOQ10 30 min before cessation of ischemia, and group IS/REP/NCOQ10: the procedure included a 3-hour ischemia, a 3-hour reperfusion, and 20 *µ*L (0.3 mmol/lit) of NCOQ10 30 min before cessation of ischemia. Significantly amended development of ischemia/reperfusion tissue injury was observed in animals treated with NCOQ10 compared to those of other groups (*P*=0.001). Mean values of biochemical indices were significantly higher than those observed for other groups (*P*=0.001). Significantly lower values of MDA were observed in IS/REP/NCOQ10 animals compared to those of other groups (*P*=0.001).Where ovarian tissue is exposed to ischemia, intraperitoneal administration of NCOQ10 could bear clinical benefits in diminishing ischemia-reperfusion injury.

## 1. Introduction

Life-threatening reduction in blood flow of tissue and everlasting tissue injury is ensued in mesovarium longer than normal. Congestion of adnexal venous can end up in torsion of the ovary and subsequently obstruction of the ovarian vessels [1]. Possible diagnostic and therapeutic measures are required to reserve ovarian performances to help preclude infertility in the future [2]. Once ovarian torsion is diagnosed, detorsion of the twisted adnexa and assessment of reperfusion of the tissue is required to avoid possible infertility even where cyanotic tissues are formed [2, 3].

A suggested pathogenesis of tissue injury in the course of reperfusion is buildup of the stimulated neutrophils that produce reactive oxygen species [4]. Lipid peroxidation in the cell is a common detrimental outcome of free radicals (FRs) that end up decreasing the membrane potential and cell damage. Malondialdehyde (MDA) causes severe cell injury via stimulation of polymerization and cross linking in components of the membrane [5]. FRs react with DNA and produce 8-hydroxyguanine (8-OHGua) that is the destructive product of DNA [6]. Despite the fact that production of FRs happen constantly in cells, the existence of endogenous antioxidant defense systems conserves tissues from the destructive effects of FRs [7].

Coenzyme Q10 (CoQ10) is an indispensable part for electron transport in oxidative phosphorylation of the mitochondria [8, 9]. It is an effective antioxidant, a potent membrane stabilizing agent and cofactor to produce adenosine triphosphate via oxidative phosphorylation. It has been extensively used in food industry. Recently, the rate of studies on the agent has augmented basically and clinically [8–11]. Many investigations have shown the beneficial effect of CoQ10 in different settings on tissue damage [8]. Nonetheless, no research has designed the effect of CoQ10 nanoparticles in ovary I/R injury.

Excessive production of reactive oxygen species can influence the in vitro culture of preantral follicles because they can act as second messengers and result in the opening of a nonspecific pore in the inner mitochondrial membrane, release of cytochrome c, and activation of caspase cascades ultimately resulting in apoptosis. Although excessive production of oxidative agents can cause cell damage and loss of follicular function, antioxidants can attenuate deleterious effects of oxidative stress [12, 13].

The application of particles in nanoscales in addition to facilitate the solubility also offers a well-ordered release of the agent. The physiologic properties of the peritoneal cavity that aids omission of toxic metabolites from the body has been effectively exploited to afford peritoneal dialysis in patients with end-stage renal ailment [14]. The same properties of the peritoneal membrane also offer a beneficial entryway in the body for many pharmacological mediators. One benefit could be that the agent attains beneficial effectiveness in the area of concern while lessening the systemic poisonousness. Intraperitoneal administration appears more beneficial and accessible when oral administration of an agent may result in complications. It goes without saying that transperitoneal absorption of the agent is quicker than enteral medicating [15].

The literature is poor regarding the effect of COQ10 nanoparticles on the genital organs. Therefore, the present study was dissimilar from the other investigations for adopting NCOQ10 on ischemia/reperfusion injury. Intended to investigate peritoneal effects of NCOQ10 on ischemia/reperfusion injury, a research was designed to conclude if NCOQ10 would, in detail, help keep against ischemia/reperfusion-induced ovarian damage.

## 2. Materials and Methods

### 2.1. Experimental Information

All chemicals used were of research grade, adopted as acknowledged with no any additional refinement and purchased from Sigma-Aldrich.

### 2.2. Ethical Considerations

The Guide of the National Academy of Sciences published by the National Institutes of Health (NIH Publication No. 85-23, revised 1985) was followed in the present study. The procedures adopted to clear any potential pain in the animals were approved by the Institutional Ethical Committee of the University under Research Code: DR-61536 and Ethical Code: IAU/UB-1029.

### 2.3. Preparation of COQ10 Nanoparticles (NCOQ10)

NCoQ10 were prepared via a protocol styled by others [16]. Ethanol (3.0 mL) was the solvent for 10 mg of CoQ10 and was the organic phase of the solution. The solution was relocated into 27.0 mL of double-distilled water and stirred at 14,000 rpm for 15 s (Heidolph, silence crasher, Germany). A rotary vacuum at a 408°C water bath was used after evaporation of ethanol, and then, for lyophilizing the remaining fraction, a freeze dryer was adopted. The coarse suspension was prepared using 30.0 mg of coenzyme Q10 added to 30.0 mL of cold distilled water containing 10% v/v ethanol. The coenzyme Q10 content was adjusted about 1 mg/mL.

### 2.4. Coq10 Nanoparticle Characterization

To study the structural characterization and size of the nanoparticles, a Transmission Electron Microscope (TEM) was adopted. Particle size distributions of nanoparticles were determined by dynamic light scattering (DLS) through a Zetasizer Nano ZS (Malvern Instruments Limited) particle analyzer. To ascertain chemical characteristics of COQ10 and NCOQ10, the Fourier transform infrared spectrophotometry (FTIR) (Shimadzu, FTIR-8400) analysis was adopted. Concentration of the nanoparticle was identified via turbidity. Five microliter of the particles was added to 95 microliter of Tris-EDTA buffer in a microtiter plate well (TE, containing 10 mM Tris, 1 mM EDTA, pH 8.0). Through monitoring the absorbance of the solution at 405 nm, the turbidity was identified. By means of running a NCOQ10 assay with a dilute solution of nanoparticles swelled with 40% acetonitrile and comparing the NCOQ10 released to a NCOQ10 calibration curve, the average amount of NCOQ10 encapsulated within each nanoparticle was measured. Five microliter of diluted nanoparticles were added to the plate, containing 100 microliter 40% acetonitrile. The plate was incubated with the acetonitrile for 20 min. Following the 20 min period of incubation, 10 microliter 250 *μ*g/ml apo-GDH, 40 microliter 80 mM glucose, and 50 microliter 0.5 mM DCPIP with 20 mM 82 CaCl2 were added. To acquire the estimated number of NCOQ10 molecules per particle, the number of molecules of NCOQ10 was divided by the number of nanoparticles in the assay. To identify stability of the particles, the signal generated from a sandwich assay for high concentrations of target DNA for a given lot of nanoparticles was compared to the signal previously generated by the particles. The signal generated by the controls was compared to the signal generated by the target DNA. The generated signals were from particles added to wells with no target DNA or a high concentration of noncomplementary DNA. An upsurge in the nonspecific properties, associated with the signal emitted from the sandwich assay, demonstrated modifications in the DNA coverage on the surface of the nanoparticle where no such upsurge took place. In this condition, the nanoparticles were considered stable.

### 2.5. Animal Grouping

For animal experimentation, we followed the methods in [17]. The animals were housed with an ambient temperature of (23 ± 3)°C, stable air humidity, and a natural day/night cycle for 14 days prior to experiment and within the study period. Standard rodent laboratory food and tap water were freely accessible for them. The study was designed as a double-blinded setting and modified according to a method designed by others [18]. Thirty healthy female Wistar rats 12 weeks of age and approximately 250 g were categorized into five experimental groups (*n* = 6) randomly. The reason for choosing the number of rats, the dose of ketamine and xylazine, and the dose of NCOQ10 was based on the methods in [17]. The groups were as follows:

Group SHAM: in this group, only laparotomy was performed and no ischemia and reperfusion were induced on the ovaries. Then, the ovaries were taken for further analyses.

Group ischemia: in this group, laparotomy was performed and the ovaries were accessed and a three-hour period of ischemia was performed on the ovaries and immediately the ovaries were taken for further analyses.

Group I/R: in this group, laparotomy was performed and the ovaries were accessed and a three-hour ischemia followed by a three-hour reperfusion were performed and immediately the ovaries were taken for further analyses. Two and half hours after initiation of ischemia, 20 microliter soybean oil (solvent of NCOQ10) was administered intraperitoneally (IP).

Group I/NCOQ10: in this group, laparotomy was performed and the ovaries were accessed and a three-hour ischemia was performed and immediately the ovaries were taken for further analyses. Two and half hours after initiation of ischemia, 20 microliter NCOQ10 (10 mg/kg) was administered IP.

Group I/R/NCOQ10: in this group, laparotomy was performed and the ovaries were accessed and a three-hour ischemia followed by a three-hour reperfusion were performed and immediately the ovaries were taken for further analyses. Two and half hours after initiation of ischemia, 20 microliter NCOQ10 (10 mg/kg) was administered IP.

In each group, the right ovaries were transported to a 10% formaldehyde solution for histopathological analyses and the left ovaries were prepared and then stored in a freezer at –80°C for biochemical analyses.

### 2.6. Surgery

We followed the methods in [17] for surgical procedures. Intraperitoneal ketamine-xylazine (ketamine 5%, 90 mg/kg and xylazine 2%, 5 mg/kg) were used to anesthetize animals. The guidelines of the Ethics Committee of the International Association for the Study of Pain [19] were followed. The experiments were approved by the ethical committee of the Islamic Azad University, Urmia Branch.

Uterine horns and adnexa were approached via midline incision. A vascular clamp was applied on vessels of the ovaries to induce ischemia. Following a three-hour induction of ischemia, both ovaries were excised histopathology and biochemistry. To induce ischemia/reperfusion, both ovaries underwent ischemia similarly, and at the termination of a three-hour period, the clamps were opened and a 3-hour reperfusion was sustained. The ovaries were then excised for histopathology and biochemistry.

### 2.7. Histology

We followed the methods in [17] for histological studies. 10% buffered formalin for 24 hours was used to fix the ovaries. Paraffin embedding was adopted on samples with a 5 *µ*m thickness. They were then dewaxed, rehydrated, and stained routinely with hematoxylin and eosin. In brief, ovaries were fixed in 2.5% buffered glutaraldehyde and postfixed in 2% OsO4 for 2 h, dehydrated through an ethanol series, and were next stained with hematoxylin and eosin. A light microscope was used to observe the samples.

A numerical scoring system was adopted based on the works in [20]. Congestion, bleeding, oedema, and loss of cohesion (separation of parenchymal cells along with the normal ovarian cortex and follicles) were scored from 0 to +3 according to their severity, where 0 = no pathological finding and scores of 1, 2, and 3 represent pathological findings of <33%, 33–66%, and >66% of the ovary, respectively. The scores for each parameter were summed, and the total tissue damage scores were calculated.

### 2.8. Biochemistry

For biochemical studies we followed the methods in [17]. Following a three-day period at −80°C, the enzyme activities were assessed in samples. The samples were ground with liquid nitrogen in a mortar. One half gram of the tissue was used for each group, and then, 4.5 mL of an appropriate buffer was used for treatment. An ultra-turrax homogenizer (IKA, Werke, Germany) was used to homogenize the mixture on ice for 15 minutes. By a refrigerator centrifuge at 4°C, the homogenates were filtered and centrifuged. To identify the enzymatic activities, the supernatants were used. All assessments were performed at room temperature. Estimation of superoxide dismutase (SOD) was according to the production of superoxide radicals generated by xanthine and the xanthine oxidase system, which reacts with nitroblue tetrazolium to form formazan dye [21]. Measurement of activity of SOD was carried out at 560 nm by inhibition degree of the reaction and was expressed as millimoles per minute per milligram of tissue. Using the thiobarbituric acid test, concentrations of ovarian lipid peroxidation were identified via estimation on MDA [22]. The tissue samples were rinsed with cold saline. Ten ml of 100 g/l KCl was used to homogenize the corpus mucosa. The homogenate (0.5 ml) was then added to a solution containing 2-thiobarbiturate (1.5 ml of 8 g/l), acetic acid (1.5 ml of 200 g/l), sodium lauryl sulfate (0.2 ml of 80 g/l), and distilled water (0.3 ml). The mixture was incubated at 98°C for one hour. N-butanol:pyridine 5 ml (ratio: 15: l) was then added. The mixture was vortexed for 1 min and centrifuged for 30 min at 4000 rpm. Using a spectrophotometer, the absorbance of the supernatant was measured at 532 nm. Through 1,1,3,3-tetramethoxypropane, the standard curve was obtained. The method of Lawrence and Burk was followed to measure GPO activity [23]. Supernatant was used for GPO measurement following tissue homogenization. The mixture was incubated after the addition of KH2PO4, EDTA, GSH, B-NADPH, NaN3, and GR. As soon H2O2 was added, the chronometer was turned on and the absorbance at 340 nm was recorded for 5 min every 15 sec. Habig and Jakoby methods were followed to determine GST activity [24]. Enzyme activity was determined in a 4 ml cuvette containing 30 mM GSH, 30 mM 1-chloro-2,6-dinitrobenzene, 0.1 M PBS (pH: 6.5), and tissue homogenate at 340 nm by using a spectrophotometer.

### 2.9. Statistical Analysis

Statistical analyses were conducted by PASW 18.0 (SPSS Inc., Chicago, IL, USA). Through examining the residual plot, model assumptions were valued. Repeated measures and a factorial ANOVA with two between-subject factors were adopted to analyze the results. In order to survey the effect of time and treatments, the Bonferroni test for pairwise comparisons was adopted. The values were expressed as means ±SD. The differences were set at *P* < 0.05.

## 3. Results

### 3.1. NCOQ10 Nanoparticle Synthesis

Through making a lipophilic NCOQ10 salt with TDMAC, the NCOQ10 was formed into the hydrophobic particles. The NCOQ10 solution was added following dissolving the TDMAC in chloroform. Prior to extraction, the water layer was a reddish orange color. The chloroform layer turned into pinkish purple color, and the water layer began to become colorless following shaking to mix. This indicated that the NCOQ10 was successfully extracted into the organic layer. Nitrogen was used to dry the organic layer, and then, the salt was dissolved in a methanol/acetonitrile mixture in which the particles were added. To load the NCOQ10, the acetonitrile helped swell the particles. The excess NCOQ10 loading solution was removed, and then, water was added. This caused the particle surface to harden, and entrapping of NCOQ10 occurred. Any excess NCOQ10 from the solution was subsequently washed (Table 1).

### 3.2. Characterization of COQ10 Nanoparticles

**Figure 1:**
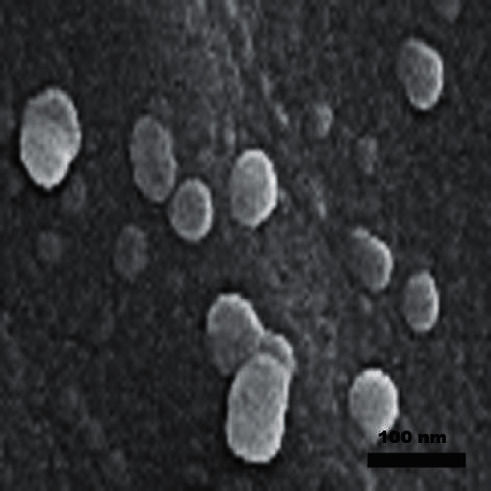
TEM micrograph of nanoparticles.

**Figure 2:**
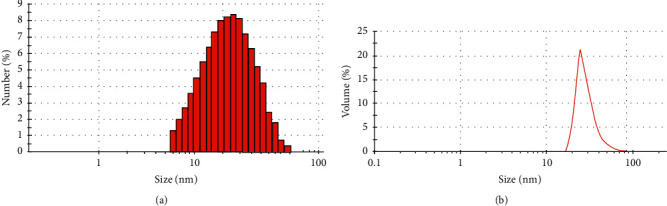
(a) Size distribution of the nanoparticles based on numbers. (b) Size distribution of the nanoparticles based on volumes.

**Figure 3:**
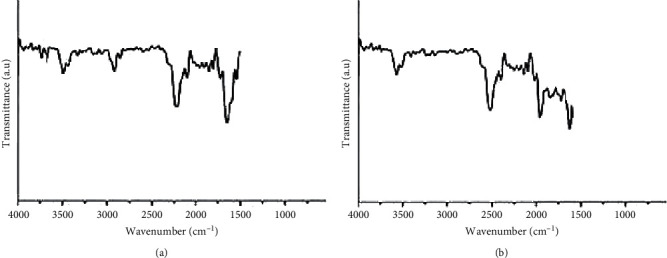
FTIR spectrum of (a) NCOQ10 and (b) NCOQ10. No significant variation between chemical properties of both particles was observed.

Several techniques were adopted to characterize the products in our study. Images of the TEM and Zetasizer Nano ZS particle analyzer are shown in Figures 1 and 2, respectively. The cerium oxide nanoparticles showed homogeneity in the composite. The particles size and volume of the cerium oxide nanoparticles were less than 100 nm approved by size distribution analyses. FTIR analysis of the NCOQ10 and NCOQ10 is depicted in Figure 3. No significant variations between chemical properties of NCOQ10 and NCOQ10 were observed using FTIR.

**Figure 4:**
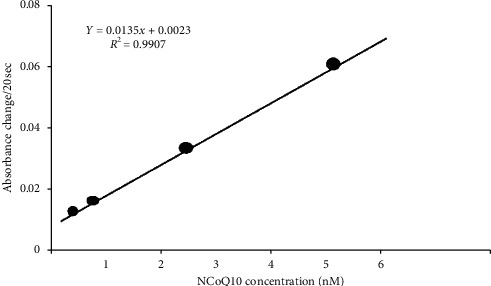
Free NCOQ10 dose response in the reconstitution assay where the acetonitrile was 40%.

**Figure 5:**
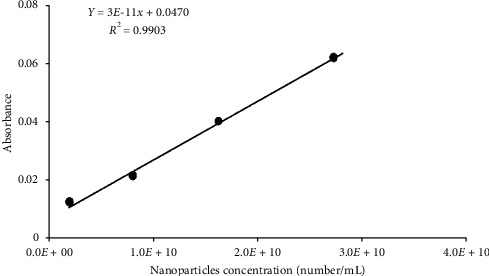
Calibration curve of turbidity to identify concentration of nanoparticles.

Running the reconstitution assay with a dilute solution of nanoparticles confirmed encapsulation of NCOQ10. The NCOQ10 was released from the nanoparticles after swelling by acetonitrile and then was accessible for reconstitution with apo-GDH. The release of NCOQ10 was enumerated using a calibration curve of NCOQ10, Figure 4. By the nanoparticle calibration curve, the total number of particles present in the solution was enumerated. The generation was according to particle turbidity, Figure 5. The number of molecules of NCOQ10 within the particle interior was identified by dividing the number of NCOQ10 molecules by the number of particles present.

### 3.3. Histology

**Figure 6:**
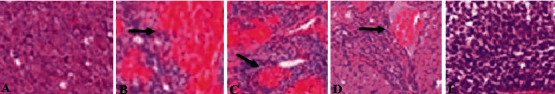
Representative histologic micrographs of the ovarian samples in SHAM, IS, IS/REP, IS/NCOQ10, and IS/REP/NCOQ10 groups. (a) Normal secondary follicles with compact stroma between them. (b) Many follicles in various stages of development are observed. Edematous ovarian stroma and multiple dilated congested blood vessels with some areas of hemorrhage are observed. (c) Ovarian follicles in various stages of development with edema in the stroma and hemorrhage are observed. (d) Many follicles in various stages of development are observed. There are edematous ovarian stroma and multiple dilated congested blood vessels with some areas of hemorrhage. (e) Normally appearing ovarian tissue with preserved healthy follicles in various stages of development with mild edema or hemorrhage is seen.

**Figure 7:**
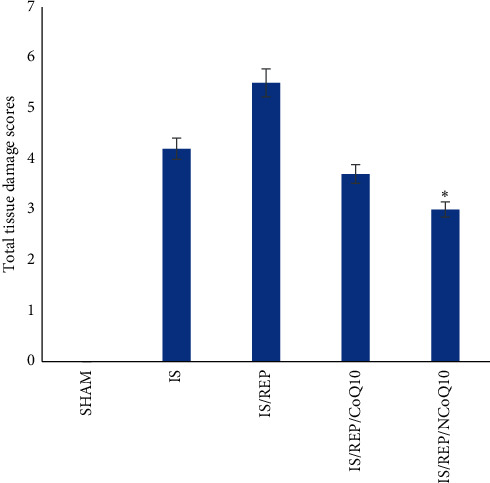
Total tissue damage scores of SHAM, IS, IS/REP, IS/NCOQ10, and IS/REP/NCOQ10 groups. Data are expressed as mean ± SD. ^*∗*^*P* < 0.05 vs. IS, IS/REP, and IS/COQ10 groups.

The histologic structure of the ovarian tissue in the SHAM animals was observed to be normal. Condensed hemorrhage and severe vascular congestion with degenerative and necrotic changes in many of the cells were observed in tissue samples in the ischemia group. Histopathological alterations of condensed hemorrhage and infiltration of inflammatory cells with degenerative and apoptotic cells were observed in ovarian tissues in the IS/REP group. Polymorphonuclear leukocytes (neutrophils) were prevailing cell types. Overall histological and cellular outlines of the tissues were not observed normal in the IS/REP/COQ10 group; in contrary, mild vascular congestion and edema were observed. Only a slightly mild hemorrhage was observed around the ovarian follicles in the IS/REP/NCOQ10 group. The general histologic outline of the ovarian tissue in the IS/REP/NCOQ10 group was normal. Except for a slightly mild inflammation, vascular congestion, and edema, no important pathologic findings were observed in the structural level in the IS/REP/NCOQ10 group, Figure 6. Total tissue damage scores were significantly different among the groups. The IS/REP/NCOQ10 group showed a significantly more improved score compared to IS, IS/REP, and IS/REP/COQ10 groups (*P* < 0.001), Figure 7.

### 3.4. Biochemistry

**Figure 8:**
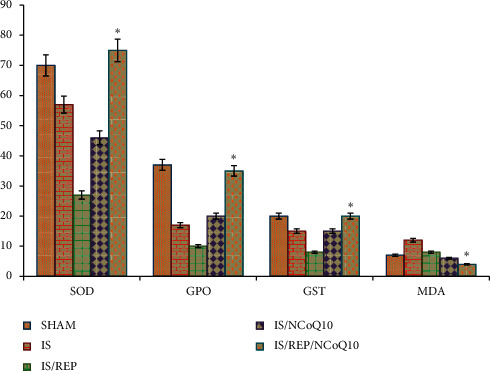
Bar graph depicts comparison of SOD, GPO, GST, and MDA activities in the ovarian samples in experimental groups. Data are expressed as mean ± SD. ^*∗*^*P* < 0.05 vs. IS, IS/REP, and IS/COQ10 groups.

In *I* and *I*/*R* groups, the mean values of SOD were diminished. NCOQ10 administered intraperitoneally inverted the trend and augmented the SOD activity in the assessed samples. The mean value of superoxide dismutase activity in the IS/REP/NCOQ10 group was significantly higher than that of the other experimental groups (*P*=0.001). Significant augmentation was observed in the level of mean value of MDA in the IS/REP group (*P*=0.001). NCOQ10 administered intraperitoneally significantly diminished the level of MDA in ovarian tissues in the IS/REP/NCOQ10 group (*P*=0.001). NCOQ10 administered intraperitoneally significantly augmented the level of GPO in ovarian tissues of the IS/REP/NCOQ10 group (*P*=0.001), Table 1. NCOQ10 administered intraperitoneally ended up in a significant increase in the level of GST in ovarian tissues of the IS/REP/NCOQ10 group (*P*=0.001), Figure 8.

## 4. Discussion

The present study was aimed to investigate if intraperitoneal administration of NCOQ10 could be of benefit in the prevention of ovarian damage in ischemia/reperfusion conditions in rat ovaries. It was found to have advantageous properties. Histopathology and biochemistry evaluations were conducted in all groups. Histopathological, edema, vascular congestion, hemorrhages, and leukocyte infiltration parameters were adopted. Histologically, ischemia, ischemia-reperfusion, and intraperitoneal NCOQ10 applications on tissues were evaluated. Findings of our study indicated that oxidative stress level was in consistency with injury to the tissue. For evaluation of the state of vascular congestion, hemorrhages, leukocyte infiltration, and cell edema histopathological parameters were adopted [3]. The findings of the present study revealed that there were a mild vascular congestion, hemorrhages, leukocyte infiltration, and cell edema in the NCOQ10-treated animals.

A high level of reactive oxygen species (ROS) and low antioxidant activity in the follicular fluid have resulted in a reduced pregnancy outcome. The suppression of ROS by antioxidants reversibly inhibits the resumption of meiosis in rat oocytes in vitro. The probable mechanism underlining these effects of ROS is linked with the stimulation of 5′ adenosine monophosphate-activated protein kinase (AMPK) and/or the Ca2+-mediated pathway that induces meiotic resumption from diplotene arrest in mammalian oocytes [13]. Overproduction of ROS can affect the in vitro culture of preantral follicles because they can act as second messengers and lead to the opening of a nonspecific pore in the inner mitochondrial membrane, release of cytochrome c, and activation of caspase cascades ultimately resulting in apoptosis. Although overproduction of oxidative agents can cause cell damage and loss of follicular function, antioxidants can attenuate deleterious effects of oxidative stress [13].

In our findings, mean values of superoxide dismutase in ovarian tissue were measured and compared in the experimental groups. The conversion of superoxide-free radical into hydrogen peroxide and molecular oxygen is catalyzed by superoxide dismutase. Free radicals are neutralized by superoxide dismutase and endogenous antioxidant. This protects tissues from the detrimental effects of free radicals and active oxygen species [25]. In our findings in the NCOQ10-treated animals, mean values of superoxide dismutase were augmented in comparison with that in the *I*/*R* group and ovarian tissue was secured against ischemia-reperfusion injury via intraperitoneal administration of NCOQ10. Malondialdehyde, a lipid peroxidation product, is produced due to the peroxidation of fatty acids with three or more double bonds. Cross linking of membrane components is ensued by malondialdehyde that ends up in undesirable consequences such as alterations in ion permeability and enzyme activity via disturbing the ion exchange through the cell membranes [26]. Malondialdehyde mean values in our findings were found to be far lesser in the NCOQ10-treated animals compared to those in other groups. Protection of the tissues against ischemia-reperfusion injury in NCOQ10-treated animals could be concluded. Activity of glutathione peroxidase is significantly diminished in tissues undergoing oxidative-stress-related settings such as ischemia-reperfusion injury [27]. Glutathione peroxidase depollutes the radicals of hydrogen peroxide that are produced in the cell via conversion to water and avoids the production of more toxic mediators from radicals of hydrogen peroxide [28]. NCOQ10 administration, in our findings, resulted in a significant augmentation in glutathione peroxidase activity. Glutathione S-transferase binds foreign substances to the–SH group of cysteine in glutathione, neutralizes the electrophilic regions, and guards the cells from the detrimental impacts of foreign substance regions [29]. Several works demonstrated that the agents with antioxidant or anti-inflammatory properties (Losartan) could be of benefit in diminution of ischemia-reperfusion damage in ovarian and testicular tissues [30].

It has been demonstrated that glutathione S-transferase activity is suppressed in oxidative tissue damage stimulated by ischemia [29]. In agreement, the results of the present study revealed that administration of NCOQ10 significantly augmented glutathione S-transferases activity in ovarian tissue.

Advantageous impact of controlled reperfusion to avoid ovarian tissue damage has been reported. Ischemia/reperfusion injury still remains to be a thoughtful issue in practice. Principally, premature diagnosis and treatment of ovarian torsion plays a vital role to offer urgent protection against life-threatening complications from ischemia and to preclude future infertility [31].

It has been documented that potent antioxidants such as Coenzyme Q10 (CoQ10) that directly scavenges reactive oxygen species and indirectly regenerates cellular vitamin E and C bear ability to diminish lipid peroxidation in membranes of cells [32]. CoQ10 is an outstanding endogenous lipid-soluble and vitamin-like agent that potently carries proton-electron in the chain of mitochondrial electron transport and accordingly supplies energy (ATP) to cell [11, 33]. Moreover, CoQ10 bears hepatoprotective effects against xenobiotic-associated cellular injury and oxidative stress in several cases such as acid salicylic acid, statins, tetrachloride carbon, doxorubicin, and thioacetamide [34–36].

CoQ10 bears significant lipid solubility and low bioavailability that limit its clinical applications, despite having a protective role against toxicities induced by xenobiotics. Therefore, for clinical applications, higher doses are required to be administered orally [37]. Others have achieved noticeable therapeutic efficiency in humans only through the long-term and high-dose CoQ10 administration that draws attention of researchers to try to find novel strategies for overcoming the limitations [16].

It has been reported that CoQ10 nanoparticles bear antioxidant properties and via these characteristics significantly ameliorated the oxidative stress generation and improved the histological alterations in kidney tissue of rats; hence, it was concluded that CoQ10 nanoparticles had the potential to reverse the oxidative stress in rat kidneys suggesting their therapeutic potential [38].

Although in the present study the outcomes were promising, the study period was relatively short; therefore, more long-term studies are required to assess outcomes of systemic administration of COQ10 nanoparticles on ischemia-reperfusion injury on the ovaries in rat that remained unknown. These could be regarded as limitations of our study.

Substances are administered by a wide variety of routes. A crucial issue to determine the route selected is whether the agent is being administered for a local or systemic effects, enteral, or parenteral. The highest bioavailability of substances is reached in parenteral administration methods, and that is because these methods prevent the first-pass effect of hepatic metabolism that takes place commonly in oral administration of chemicals and therapeutics [39]. Intraperitoneal administration appears to be more operative and obtainable where oral administration of an agent may cause difficulties. It goes without saying that transperitoneal absorption of therapeutics is much quicker than enteral administration because it is time saving that is very vital in emergency conditions such as ovarian torsion [39].

It could be concluded that the findings of histology results acquired from the experimental groups of our study were in agreement with the findings of biochemistry demonstrating that intraperitoneal administration of NCOQ10 could be of benefit in diminishing ischemia-reperfusion injury in ovarian tissue exposed to ischemia. Regarding clinical usage of the transperitoneal absorption of the NCOQ10 that is quicker than its enteral administration, it could be well-thought-out in clinical practice where ovarian torsion is the case and ovarian functions must be resumed as early as possible to preserve and avoid future infertility. The present work showed that intraperitoneal administration of NCOQ10 could recover ischemia-reperfusion damage in ovarian tissue exposed to ischemia. Dose-dependent studies remain to be conducted to get the maximum effect of the NCOQ10 on ischemia-reperfusion damage in ovarian tissue.

## Figures and Tables

**Table 1:** Average size and number of the molecules. The data are expressed as mean ± SD.

Average size of the molecules (nm)	83 ± 7
Average number of CoQ10 nanoparticles	34000 ± 750000
Stability of CoQ10 nanoparticles	>15 days

## Data Availability

The data used to support the findings of this study are available from the corresponding author upon request.
